# Butyrylcholinesterase (BChE) downregulation in taxane resistance: implications for prostate cancer

**DOI:** 10.55730/1300-0152.2743

**Published:** 2025-03-26

**Authors:** Buse CEVATEMRE, Arda IŞIKLAR, İpek BULUT, Ezgi KARYEMEZ, Hamzah SYED, Ceyda AÇILAN

**Affiliations:** 1 Division of Medical Biology, Faculty of Medicine, Koç University, İstanbul, Turkiye; 2 Research Center for Translational Medicine, Koç University, İstanbul, Turkiye; 3 Graduate School of Health Sciences, Koç University, İstanbul, Turkiye

**Keywords:** Prostate cancer, drug resistance, chemotherapy, taxane

## Abstract

**Background/aim:**

Despite advancements in chemotherapeutic strategies, drug resistance remains a major barrier to effective cancer treatment. While primary prostate cancers (PC) can be treated with surgery and radiotherapy, treatment options for recurrent PC are limited. Upon progression to the castration-resistant (CR) phase, therapies rely on taxanes (chemotherapeutic drugs) like docetaxel (Dtx) and cabazitaxel (Cbz); however, resistance to taxanes is common in CRPC, highlighting the importance of identifying underlying molecular mechanisms or targets in resistant cells.

**Materials and methods:**

In transcriptome (RNA sequencing) analyses comparing taxane-resistant PC cells (resistant to both Dtx and Cbz) with parental (nonresistant, sensitive) cells, butyrylcholinesterase (*BChE*) was identified as the most significantly downregulated gene. Although low serum BChE levels have been documented in various cancers, its role in chemotherapy resistance remains unclear. To address this gap, we validated its expression in taxane-resistant CRPC lines and manipulated *BChE* levels in both parental and resistant cells via lentiviral overexpression or depletion using shRNA and gRNA (CRISPR-Cas9), to assess its impact on taxane resistance.

**Results:**

BChE suppression in parental CRPC cells conferred resistance, whereas its overexpression in taxane-resistant cells was insufficient to resensitize them. Analysis of publicly available databases showed reduced *BChE* mRNA levels in patient samples across various cancers, including PC. Additionally, The Cancer Genome Atlas (TCGA) analyses identified *BChE* as a significant posttreatment neoplasm marker in PC.

**Conclusion:**

Our study confirmed the downregulation of *BChE* in taxane-resistant CRPC cell models and established its role in conferring resistance when depleted in parental CRPC cells, highlighting its association with taxane resistance. Additionally, the identification of BChE as a posttreatment neoplasm marker, derived from data mining analyses, suggests it might serve as a biomarker for tracking disease progression in PC.

## 1. Introduction

Localized prostate cancer (PC) is primarily treated with surgery or radiotherapy. However, recurrence is frequent, often requiring castration via surgical methods or androgen deprivation therapy (ADT) due to the androgen dependence of PC. ADT provides initial benefits for 80%–90% of patients, with a median progression-free survival (PFS) ranging from 12 to 30 months (Schutz et al., 2014). Unfortunately, once the disease progresses to castration-resistant prostate cancer (CRPC), the median overall survival (OS) declines significantly to 8–16 months (Schutz et al., 2014). Microtubule-stabilizing taxanes, such as docetaxel (Dtx) (Tannock et al., 2004) and cabazitaxel (Cbz) (de Bono et al., 2010), are standard treatments for CRPC and can extend survival, although resistance frequently develops. Various processes contribute to taxane resistance in CRPC, including hyperacetylation of tubulin (Pu et al., 2024), activation of epithelial–mesenchymal transition (Marín-Aguilera et al., 2014), epidermal growth factor receptor (EGFR) signaling (Hour et al., 2015), the GR–LEDGF/p75 axis (Sanchez-Hernandez et al., 2023), GATA2-IGF2 axis (Vidal et al., 2015), ETS transcription factor (*ERG*) (Galletti et al., 2014), lncRNAs (Pucci et al., 2020), miRNAs (Wang et al., 2016; Armstrong et al., 2017), multinucleated polyploidy (Mittal et al., 2017), decreased SLCO1B3 influx transporter levels (de Morrée et al., 2016), and increased activity of drug efflux transporters (ABC family) (Lombard et al., 2017; Huang et al., 2020; Lombard et al., 2021). Among these, our previous study demonstrated that *ABCB1*, identified as the most significantly upregulated gene in transcriptomic analyses of taxane-resistant CRPC cells, plays a critical role in resistance and that genomic targeting via CRISPR can resensitize cells to taxanes (Cevatemre et al., 2024). These analyses revealed butyrylcholinesterase (*BChE*) as the most significantly downregulated gene.

BChE, an α-glycoprotein synthesized in the liver and secreted into the bloodstream, has been considered an orphan enzyme with no essential physiological role beyond metabolizing dietary or medicinal esters (Brimijoin et al., 2016). However, studies have revealed its significant role in hydrolyzing ghrelin—the hunger hormone—affecting weight gain, fat metabolism, and even emotional behaviors such as aggression and social stress (Brimijoin et al., 2016). Given its involvement in metabolic regulation and detoxification, BChE may influence cellular stress responses—processes that cancer cells frequently reprogram to support survival and therapy resistance. Low serum BChE levels have also been reported in patients with pancreatic (Klocker et al., 2020), colorectal (Morera Ocón et al., 2007), oral (Nair et al., 2017), head and neck (Chougule et al., 2008), non-small cell lung (Shin et al., 2017), and bladder (Koie et al., 2014) cancers, with some of these studies further showing an association with overall survival and highlighting its potential prognostic value. The prognostic relevance of preoperative BChE levels in PC patients undergoing radical prostatectomy was explored (Koie et al., 2016). The study demonstrated that decreased preoperative BChE levels were independently linked to reduced biochemical recurrence-free survival (BRFS), suggesting that BChE could act as a significant prognostic biomarker in PC. Nevertheless, neither this study nor other existing research provides any insights into the relationship between BChE and chemotherapy resistance. Building on our findings of BChE downregulation in taxane-resistant cells, this study aims to further investigate the role of BChE by examining how its gene manipulation—including depletion via shRNA-mediated knockdown and CRISPR-Cas9, or overexpression—affects taxane resistance.

## 2. Materials and methods

### 2.1. RNA-sequencing and differential gene expression analysis

To characterize the transcriptional alterations associated with taxane resistance in CRPC, RNA sequencing (RNA-seq) was performed in our previous study using DU145 parental and taxane-resistant cell lines. Differential gene expression analysis was conducted for the following comparisons: (i) DU145-DtxR vs. parental cells and (ii) DU145-CbzR vs. parental cells. RNA extraction, library preparation, sequencing, and data analysis were carried out as previously described (Cevatemre et al., 2024). The RNA-seq dataset is available in the gene expression omnibus (GEO) repository under accession number GSE247557.

### 2.2. Construction and cloning of shRNA and gRNA expression vectors

Sequences targeting BChE mRNA (accession: NM_000055.4, GI: 1519314210) were used to design shRNA constructs. The design followed the Broad Institute’s shRNA design rules, creating 19–21 nucleotide hairpin structures. BLASTn analysis and intrinsic scoring ensured high specificity and minimized off-target effects. The gRNA sequences were designed using Benchling. Oligonucleotides were dissolved in DNase-free water (100 μM), annealed by incubation at 37 °C for 30 min, heated to 95 °C and gradually cooled to 25 °C, then phosphorylated with T4 PNK (M0201, New England Biolabs, Ipswich, MA, USA) according to the manufacturer’s instructions. The pLKO.1-puro plasmid (8453, Addgene, Cambridge, MA, USA, 2 μg) was linearized with EcoRI-HF (R3101, New England Biolabs, Ipswich, MA, USA) and AgeI-HF (R3552, New England Biolabs, Ipswich, MA, USA) at 37 °C for 3 h, followed by heat inactivation at 65 °C for 20 min. Meanwhile, the LentiCRISPR v2 plasmid (52961, Addgene, Cambridge, MA, USA, 2 μg) was digested with BsmBI-v2 (R0739, New England Biolabs, Ipswich, MA, USA) at 55 °C for 3 h. The digested vectors were dephosphorylated using Antarctic Phosphatase (M0289, New England Biolabs, Ipswich, MA, USA) for 30 min and purified with the NucleoSpin Gel and PCR Cleanup kit (Macherey–Nagel, 740609, Germany), both according to the manufacturer’s instructions. Purified vectors (100 ng) were ligated with annealed shRNA or gRNA oligos (1:200 dilution) using T4 DNA Ligase (Jena Bioscience, EN-149, Germany) in a 10 μL reaction. Ligation was performed at 25 °C for 2 h before transformation. For transformation, 10–50 ng of ligation product was mixed with 50 μL of competent cells on ice for 30 min. Heat shock was performed for 45 s, followed by immediate cooling on ice for 2 min. The cells were incubated at 37 °C for 45 min with shaking. Finally, the culture was plated on antibiotic-containing agar plates and incubated at 37 °C overnight. The shRNA and gRNA sequences used are detailed in Table 1.

### 2.3. Lentivirus production and cell transduction

Cloned plasmids were cotransfected into HEK293T cells with psPAX2 (12260, Addgene, Cambridge, MA, USA, 2250 ng) and VSV.G (14888, Addgene, Cambridge, MA, USA, 250 ng) using FuGENE6 transfection reagent (E2691, Promega, Madison, WI, USA). After 24 h, the culture medium was replaced, and lentivirus-containing supernatants were collected over three days, filtered, and subsequently precipitated using PEG (P2139, Sigma-Aldrich (MO, USA)). Viruses were further precipitated by centrifugation, diluted with phosphate-buffered saline (PBS), aliquoted, and stored at −80 °C. Lentiviral particles were used to infect parental cells at a low multiplicity of infection (MOI) (0.5–1) in the presence of 8 μg/mL protamine sulfate (P3369, Sigma-Aldrich (MO, USA)). Infected cells were selected by treatment with puromycin (P8833, Sigma-Aldrich (MO, USA)) at 1 μg/mL for 3–4 days. The expected reduction in BChE gene expression was validated by qRT-PCR.

### 2.4. Stable overexpression of BChE in taxane-resistant cells

The *BChE* gene, cloned into the pLenti6.3/V5-DEST plasmid, was purchased from DNASU plasmid repository (HsCD00943561, Tempe, AZ, USA). Lentiviral particles were produced as described above, and used to transduce taxane-resistant cells, resulting in stable overexpression of BChE.

### 2.5. qRT-PCR analysis

RNA isolation was performed using the Quick-RNA MicroPrep Kit (R1051, Zymo Research, Irvine, CA, USA) according to the manufacturer’s protocol. RNA concentration (ng/μL) was measured using the NanoDrop 2000 spectrophotometer (Thermo Fisher Scientific, Waltham, MA, USA). cDNA synthesis was carried out using 500 ng of RNA and the M-MLV reverse transcriptase enzyme (28025013, Invitrogen, Carlsbad, CA, USA). Quantitative real-time PCR (qRT-PCR) analysis of mRNAs was performed using the PikoReal Real-Time PCR system (Thermo Scientific, Waltham, MA, USA). Gene expression levels were normalized to β-actin expression, and relative fold changes were calculated using the 2^−ΔΔCt^ method. Amplification specificity was confirmed by analyzing melting curves. The primers and sequences used in this study are listed in Table 1.

### 2.6. SDS-PAGE and Western blot (WB)

Cell lysis was performed using RIPA buffer (RIPA-100, EcoTech Biotechnology, Türkiye) by incubating the cells for 30 min at +4 °C in the dark. Following lysis, samples were centrifuged at 16,000 × *g* for 15 min at +4 °C, and the supernatant was collected for protein concentration measurement using the bicinchoninic acid method (23225, Thermo Fisher Scientific, Waltham, MA, USA). Proteins (20–40 μg) were loaded onto a 4%–15% Tris-glycine SDS gel (4561025, Bio-Rad, Hercules, CA, USA) and electrophoresed at 80 V for 1–1.5 h. Separated proteins were transferred onto a polyvinylidene fluoride (PVDF) membrane (1620177, Bio-Rad, Hercules, CA, USA) using a BioRad transfer system. The membrane was blocked with 5% nonfat dry milk (1706404, Bio-Rad, Hercules, CA, USA) for 1 h at room temperature (RT), and washed three times for 5 min each with Tris-buffered saline with Tween 20 detergent (TBST). The membrane was incubated overnight at +4 °C with a primary BChE antibody (23854-1-AP, Proteintech, Chicago, IL, USA). After three 5-min washes with TBST, the membrane was incubated for 1 h at RT with a secondary antibody. Protein bands were visualized using the Odyssey XF imaging system (LI-COR Biosciences, Lincoln, NE, USA).

### 2.7. Sulforhodamine B (SRB) viability assay

DU145-P cells (4 × 10^3^ cells/well) and 22Rv1-P cells (1 × 10^4^ cells/well) were seeded into 96-well plates. After 24 h, cells were treated with taxanes and incubated for 72 h. Following incubation, 50 μL of 50% (w/v) trichloroacetic acid (T6399, Sigma-Aldrich (MO, USA)) was added to each well (final concentration 10%) and incubated at +4 °C for 1 h to fix the cells. The wells were washed five times with distilled water, and 50 μL of sulforhodamine B (SRB) dye (sc-253615, Santa Cruz Biotechnology, Santa Cruz, CA, USA) was added for staining. After 30 min at RT, wells were washed five times with 1% (v/v) acetic acid (ISOLAB, 901.013, Göttingen, Germany). Bound dye was dissolved by adding 150 μL of 10 mM Tris base (T6687, Sigma-Aldrich (MO, USA)), and absorbance (Abs) was measured at 570 nm. Docetaxel (01885) and Cabazitaxel (SML2487) were purchased from Sigma-Aldrich (MO, USA).

### 2.8. Statistical analysis

All statistical analyses were performed using GraphPad Prism 10 software (GraphPad Software Inc.; San Diego, CA, USA). Data are presented as mean ± standard error of the mean (SEM). The statistical significance tests used for analysis are specified in the figure legends. Each experiment was performed with at least duplicate samples, and the corresponding p-values are detailed in the figure legends.

## 3. Results

### 3.1. *BChE* is significantly downregulated in taxane-resistant CRPC cells

**Figure 1:**
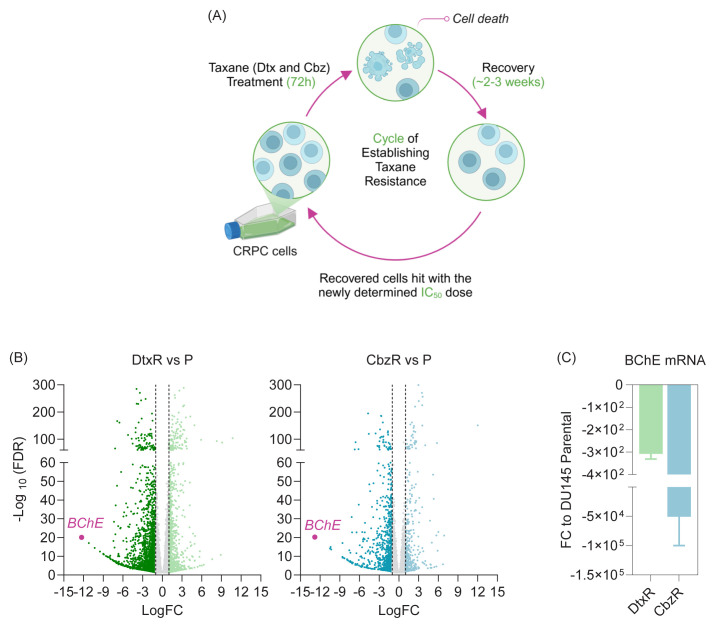
BChE downregulation in taxane-resistant CRPC models. **(A)** Establishment of taxane-resistant cell lines, as illustrated using BioRender. **(B)** BChE downregulation is visualized on volcano plots comparing cell lines resistant to docetaxel (Dtx) and cabazitaxel (Cbz) to the parental DU145 CRPC line. **(C)** The RNA sequencing results were validated by qRT-PCR using newly isolated RNA. **DtxR:** Docetaxel-resistant, **CbzR:** Cabazitaxel-resistant, **P:** Parental.

Taxane resistance was established by subjecting CRPC cells to repeated cycles of treatment with docetaxel (Dtx) or cabazitaxel (Cbz) for 72 h, followed by a recovery period of approximately 2 to 3 weeks. Recovered cells were subsequently treated with the newly determined IC_50_ dose, as illustrated in Figure 1A. This cycle continued for approximately 8 to 12 months, until the cells no longer responded to high doses of taxanes (e.g., 125 nM). Hereafter, docetaxel-resistant cells are referred to as DtxR, and cabazitaxel-resistant cells as CbzR. Detailed characterization of these cells was presented in our previous study (Cevatemre et al., 2024), which showed that they neither underwent cell death nor G_2_/M arrest upon taxane treatment—the primary mechanism of action of taxanes (Gelmon, 1994). Transcriptome analyses revealed that *BChE* was the most significantly downregulated gene—with the highest fold change (FC)—in taxane-resistant CRPC cells compared to parental cells (Figure 1B). The analysis for DtxR cells showed significant downregulation of *BChE* with a Log_2_FC of −12.3, a highly significant p-value (p = 4.35 × 10^−22^), and a false discovery rate (FDR) of (8.81 × 10^−21^). Similarly, CbzR cells exhibited a Log_2_FC of −12.8, with a highly significant p-value (p = 1.33 × 10^−22^) and FDR (5.61 × 10^−21^). These findings were further validated by qRT-PCR (Figure 1C).

### 3.2. Taxane responses of BChE-depleted parental cells

**Figure 2:**
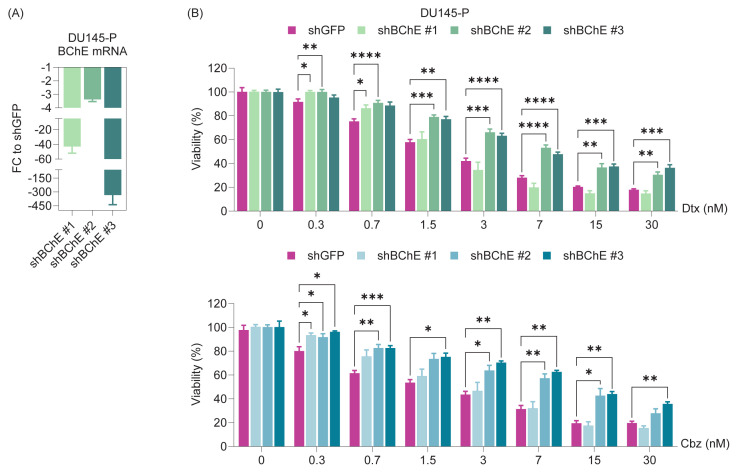
BChE depletion in DU145 parental cells and response to taxanes. **(A)** The BChE gene in parental cells was silenced using shRNA sequences (shBChE #1/#2/#3) cloned into a lentiviral vector, and knockdown efficiency was validated by qRT-PCR in stable cell lines. **(B)** The taxane response in shBChE-recieved cells was assessed using the SRB viability assay after 72 h. Statistical significance levels are indicated as *: p < 0.05, **: p < 0.01, ***: p < 0.001, ****: p < 0.0001, compared to the control group (shGFP). **P:** Parental.

Upon stable and successful suppression of the *BChE* gene (Figure 2A), two out of three shRNA constructs expressed in DU145 parental cells were found to confer reduced sensitivity to both taxanes (Dtx and Cbz), suggesting the acquisition of resistance (Figure 2B).

**Figure 3:**
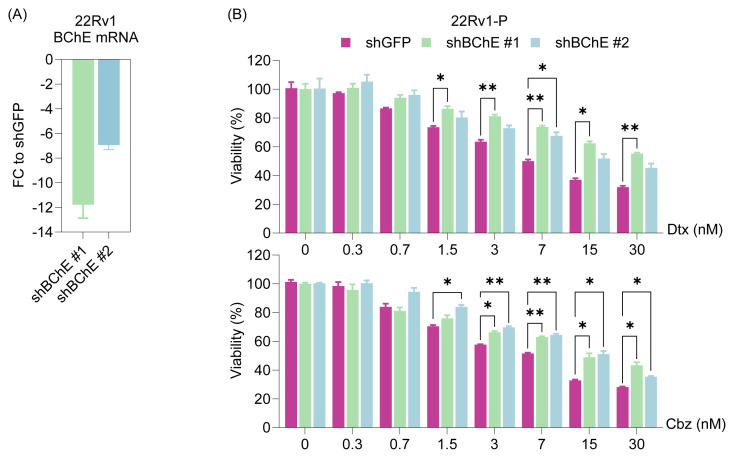
BChE depletion in 22Rv1 parental cells and response to taxanes. **(A)** The BChE gene in parental cells was silenced using shRNA sequences (shBChE #1 and #2) cloned into a lentiviral vector, and knockdown efficiency was validated by qRT-PCR in stable cell lines. **(B)** The taxane response in shBChE-recieved cells was assessed using the SRB viability assay after 72 h. Statistical analysis was performed using two-way ANOVA in GraphPad Prism. Statistical significance levels are indicated as *: p < 0.05, **: p < 0.01 compared to the control group (shGFP). **P:** Parental.

To ensure reproducibility, we extended this analysis to another CRPC cell line (22Rv1), where BChE knockdown also led to significantly increased IC_50_ values for both taxanes (Figure 3; Table 2). In particular, 22Rv1-shBChE#1 cells exhibited a dramatic increase in Dtx IC_50_ from 7.50 nM to 40.76 nM, while Cbz IC_50_ increased from 7.66 nM to 14.38 nM. These consistent findings across two independent cell models highlight the functional relevance of BChE in the cellular response to taxane treatment.

**Figure 4:**
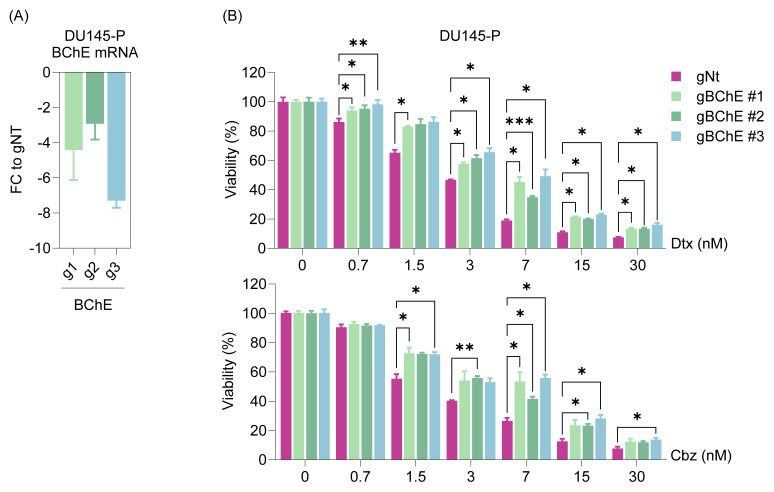
BChE depletion by CRISPR-Cas9 in DU145 parental cells and response to taxanes. **(A)** The BChE gene in parental cells was depleted using gRNA sequences (gBChE#1/#2/#3) cloned into a lentiviral vector, and knockdown efficiency was validated by qRT-PCR in stable cell lines. **(B)** The taxane response in gBChE-received cells was assessed using the SRB viability assay after 72 h. Statistical analysis was performed using two-way ANOVA in GraphPad Prism. Statistical significance levels are indicated as *: p < 0.05, **: p < 0.01 ***: p < 0.001 compared to the control group (shGFP). **P:** Parental.

We aimed to utilize CRISPR-Cas9 to validate the findings by increasing methodological diversity and minimizing potential off-target effects associated with genomic manipulation, thereby enhancing the biological relevance of the results. BChE targeting via gRNA produced similar results, which were slight but statistically significant, as shown in Figure 4 and Table 2. For example, in DU145-gBChE#3 cells, the IC_50_ for Dtx increased from 2.74 nM (gNt control) to 6.81 nM, while the IC_50_ for Cbz increased from 2.03 nM to 8.69 nM. These findings further support the role of BChE loss in conferring taxane resistance.

### 3.3. Taxane responses of resistant cells following BChE overexpression

**Figure 5:**
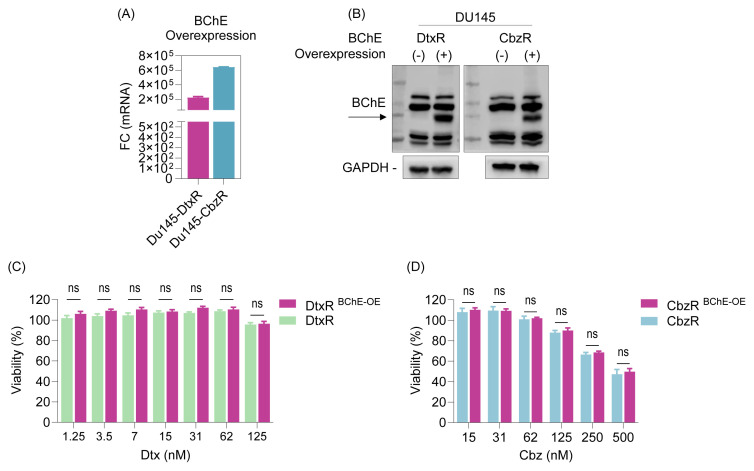
Overexpression of BChE in taxane-resistant cells and response to taxanes. The BChE gene was overexpressed in taxane-resistant cells using a lentiviral system, and expression efficiency was validated in stable cell lines through **(A)** qRT-PCR and **(B)** WB. **(C)** The taxane response in BChE-overexpressing DtxR cells was assessed after 72 h using the SRB assay and in **(D)** CbzR cells using the CTG viability assay. Statistical analysis was performed using two-way ANOVA in GraphPad Prism. Ns (nonsignificant) indicates no statistical significance compared to the control group.

To determine whether BChE reexpression could restore taxane sensitivity in resistant cells, we generated stable BChE-overexpressing cell lines derived from DtxR and CbzR models. Overexpression efficiency was confirmed by qRT-PCR and Western blot analysis, both of which demonstrated a significant increase in BChE expression levels compared to vector controls (Figures 5A and 5B). Despite robust BChE overexpression, no significant changes in taxane sensitivity were observed, indicating that BChE overexpression alone is insufficient to reverse taxane resistance (Figure 5C).

**Figure 6:**
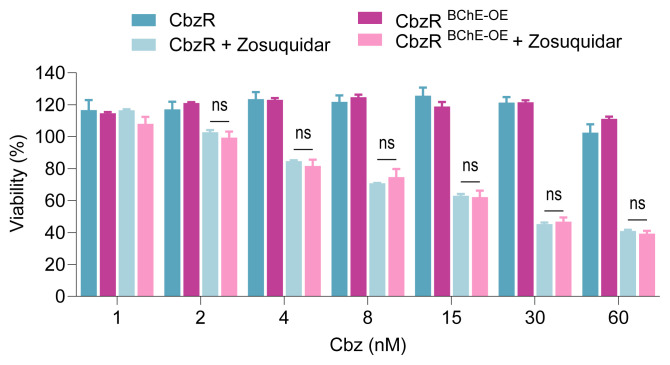
The taxane response in BChE-overexpressing and nonoverexpressing CbzR cells was assessed in the presence and absence of the ABCB1 inhibitor Zosuquidar (125 nM) using the CTG viability assay after 72 h. Statistical analysis was performed using two-way ANOVA in GraphPad Prism software. Ns (nonsignificant) indicates no statistical significance compared to the control group.

As demonstrated in our previous study, ABCB1 plays a significant role in taxane-resistant cells, and its suppression leads to resensitization (Cevatemre et al., 2024). We considered the possibility that combining BChE overexpression with ABCB1 inhibition might promote resensitization. However, this approach was also found to be insufficient to reverse taxane resistance, as BChE overexpression did not confer additional benefit compared to ABCB1 inhibition alone (Figure 6). These results suggest that while BChE loss is associated with taxane resistance, its reintroduction does not significantly affect drug sensitivity, possibly due to dominant resistance mechanisms mediated by ABCB1 or other compensatory pathways.

### 3.4. Evaluation of BChE expression in patients

**Figure 7:**
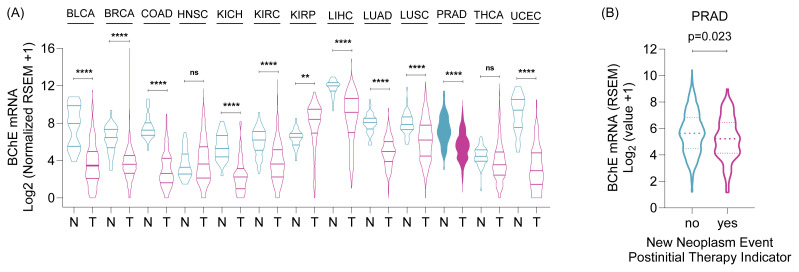
BChE mRNA expression in cancer types and clinical relevance in prostate adenocarcinoma. **(A)** BChE mRNA expression levels in normal **(N)** and tumor **(T)** tissues across various cancer types are shown. Data were obtained using the Wanderer tool (http://maplab.imppc.org/wanderer/). Statistical significance was determined using one-way ANOVA, with significance levels indicated as **: p < 0.01 and ****: p < 0.0001, while Ns (nonsignificant) denotes no statistical significance compared to the normal group. **Cancer types:** BRCA (breast cancer), COAD (colon adenocarcinoma), ESCA (esophageal carcinoma), HNSC (head and neck squamous cell carcinoma), KICH (kidney chromophobe), KIRC (kidney renal clear cell carcinoma), KIRP (kidney renal papillary cell carcinoma), LIHC (liver hepatocellular carcinoma), LUAD (lung adenocarcinoma), LUSC (lung squamous cell carcinoma), OV (ovarian serous cystadenocarcinoma), PAAD (pancreatic adenocarcinoma), PCPG (pheochromocytoma and paraganglioma), PRAD (prostate adenocarcinoma), READ (rectum adenocarcinoma), SKCM (skin cutaneous melanoma), STAD (stomach adenocarcinoma), THCA (thyroid carcinoma), UCEC (uterine corpus endometrial carcinoma). **(B)** The relationship between BChE mRNA expression and “new neoplasm event post initial therapy indicator” in prostate adenocarcinoma (TCGA, PanCancer Atlas) is shown. Data were accessed via the cBioPortal platform, and statistical significance was determined using a t-test.

To assess the clinical relevance of BChE expression, we analyzed publicly available RNA-seq datasets from the Wanderer platform, comparing BChE mRNA levels between PC tissues and normal prostate tissues (Figure 7A). Expression of BChE was significantly lower in PC tissues (p < 0.001) across multiple cancer types, suggesting that BChE downregulation may be linked to PC development or progression. Consistently, lower BChE mRNA levels (p = 0.023) in PC patients with new tumor development following initial treatment highlight the potential importance of this gene in cancer progression or drug resistance (Figure 7B).

## 4. Discussion

BChE is involved in a wide range of physiological and pathological processes, and its activity varies depending on the disease context. Although it is primarily associated with lipid metabolism and fasting responses, dysregulation of BChE has also been reported in neurodegenerative diseases, systemic inflammation, and various cancers. In Alzheimer’s disease, BChE activity increases during advanced stages, while acetylcholinesterase (AChE) levels decline markedly, suggesting a compensatory mechanism in cholinergic signaling and cognitive function (Xing et al., 2021). Conversely, BChE activity is significantly reduced during acute systemic inflammation, as observed in patients compared to healthy individuals (Zivkovic et al., 2015). In cancer, the reduction of BChE parallels its decline in inflammatory conditions. In line with these observations, our results showed a marked downregulation of *BChE* in taxane-resistant PC cells. This raises the possibility that its suppression may be linked to an inflammatory microenvironment contributing to therapy resistance.

The aim of this study, driven by the identification of *BChE* as the most significantly downregulated gene in taxane-resistant cells (Figure 1), was to evaluate how cells respond to taxane treatment after BChE suppression or overexpression. Analyses revealed that BChE suppression (via shRNA and gRNA) resulted in an increase in IC_50_ values (Figures 2–4, Table 2), suggesting that BChE may have a role in drug resistance. Given that overexpression of BChE failed to resensitize resistant cells, it is evident that manipulating a single gene may not be sufficient to overcome resistance (Figure 5). This finding suggests that resistance is not likely caused by the dysregulation of a single gene, but rather by a network of molecular pathways and interactions.

In patient samples, BChE mRNA expression levels were found to be lower in PC cells compared to normal tissue (Figure 7A). This finding is consistent with previous studies (Morera Ocón et al., 2007; Chougule et al., 2008; Koie et al., 2014; Nair et al., 2017; Shin et al., 2017; Klocker et al., 2020) and supports the notion that BChE suppression in cancer cells may play a role in cancer development or progression. Reduced BChE expression was observed in tumorigenic stem cell-like PC cells compared to non-stem cells, indicating potential alterations in BChE levels during cancer progression (Gu et al., 2018). In the same study, analysis of two independent PC populations—Memorial Sloan Kettering Cancer Center (MSKCC) (n = 130) and The Cancer Genome Atlas (TCGA) Provisional (n = 490)—showed that BChE mRNA levels decreased from WHO Grade Group (WHOGG)-1 to WHOGG-3, but significantly increased in WHOGG-5 tumors. This increase was associated with reduced disease-free survival (p = 0.008), highlighting a biphasic regulation of BChE, characterized by downregulation in early-stage PC and upregulation in advanced stages. In less aggressive brain tumors, BChE activity was low to moderate, whereas in aggressive tumors, it was high. This suggests a potential link between BChE activity levels and the rate of cell growth in brain tumors, further supporting the notion of a biphasic role for BChE in tumor progression (Barbosa et al., 2001).

The prognostic value of pretreatment BChE levels in PC patients undergoing radical prostatectomy was also investigated (Koie et al., 2016). Patients with BChE levels ≥168 U/L had significantly better biochemical recurrence-free survival compared to those with BChE levels ≤167 U/L (Koie et al., 2016). In another study evaluating cholinesterase (AChE and BChE) activities and biochemical determinations in patients with PC, plasma BChE activities were found to be decreased in PC patients compared to the control group (Battisti et al., 2012). More importantly, BChE activities were significantly lower in patients with a Gleason score of seven or higher compared to other groups (Battisti et al., 2012). Additionally, the study showed reduced BChE activity in all patient groups (localized and bone metastasis) relative to controls, with the greatest reduction observed in patients with bone metastasis (Battisti et al., 2012). An additional aspect considered in this study was the impact of reduced cholinesterase activity on the hydrolysis of acetylcholine, as the authors referred to another study where acetylcholine was shown to promote the proliferation of cancer cells in lung tumors (Song et al., 2003). The authors suggested that a decrease in cholinesterase levels could lead to elevated AChE concentrations, which might trigger excessive cholinergic stimulation and contribute to increased cancer cell growth. Similarly, in later years, it was demonstrated that in hepatocellular carcinoma (HCC), AChE activated the STAT3 and AKT pathways by interacting with the androgen receptor (AR), enhancing the migration and invasion of HCC cells while inhibiting their apoptosis (Nie et al., 2013). Likewise, the release of ACh by human colon cancer cells was shown to drive autocrine proliferation (Cheng et al., 2008). Although BChE deficiency has been shown to promote adipose tissue growth (Chen et al., 2016), whether butyrylcholine levels directly stimulate growth in the context of cancer—similar to the previously mentioned role of AChE—remains an intriguing question for further investigation.

We found that patients who developed new tumors after initial treatment exhibited lower BChE mRNA levels (Figure 7B), suggesting the potential utility of monitoring mRNA levels during treatment. Indeed, the use of BChE as a biomarker could aid in assessing the efficacy of cancer treatments and tracking disease progression. For example, BChE was identified as a critical and independent prognostic factor for endometrial carcinoma (EC) patients (Liu et al., 2022). Gene set enrichment analysis linked BChE to immune pathways involved in regulating immune response, including TGF-β and PD1 blockade. Immune profiling showed a negative correlation between BChE expression and CD4+ regulatory T cells (Tregs), as well as significant associations with immune checkpoint molecules such as CD28, ADORA2A, BTNL2, and TNFRSF18. Additionally, genetic alteration analyses revealed that BChE expression is linked to tumor mutation burden. These findings suggest that BChE is a critical biomarker for EC, influencing prognosis and potentially guiding immunotherapy (Liu et al., 2022).

The reported downregulation of BChE, previously associated with prognosis, may now for the first time be linked to drug resistance. Our findings provide evidence that BChE suppression contributes to drug resistance, suggesting a potential role beyond prognosis. While these results were derived from cell models, confirming this trend in patient tissues would be essential to establish its clinical relevance. Investigating the differences in BChE expression levels between stromal and cancer cells in drug-refractory patient tissues and correlating these differences with disease progression could provide valuable insights into the role of BChE in cancer drug resistance.

## Figures and Tables

**Table 1:** BChE shRNA and gRNA sequences used in the study.

Oligo name	Sequence (5′→3′)
**shRNA**	
sh-BChE #1-F	CCGGTTGGAAATGACAGGAAATATTCTCGAGAATATTTCCTGTCATTTCCAATTTTTG
sh-BChE #1-R	AATTCAAAAATTGGAAATGACAGGAAATATTCTCGAGAATATTTCCTGTCATTTCCAA
sh-BChE #2-F	CCGGCTGACCAAGTGGTCTGATATTCTCGAGAATATCAGACCACTTGGTCAGTTTTTG
sh-BChE #2-R	AATTCAAAAACTGACCAAGTGGTCTGATATTCTCGAGAATATCAGACCACTTGGTCAG
sh-BChE #3-F	CCGGGGCTCGGGTTGAAAGAGTTATCTCGAGATAACTCTTTCAACCCGAGCCTTTTTG
sh-BChE #3-R	AATTCAAAAAGGCTCGGGTTGAAAGAGTTATCTCGAGATAACTCTTTCAACCCGAGCC
sh-GFP-F	CCGGTGAATTAGATGGCGATGTTAACTCGAGTTAACATCGCCATCTAATTCATTTTTG
sh-GFP-R	AATTCAAAAATGAATTAGATGGCGATGTTAACTCGAGTTAACATCGCCATCTAATTCA
**CRISPR-Cas9**	
gBChE #1-F	CACCGAGTAAACTTTGGTCCGACCG
gBChE #1-R	aaacCGGTCGGACCAAAGTTTACTC
gBChE #2-F	CACCGAGACCTGAAAACTACCGTG
gBChE #2-R	aaacCACGGTAGTTTTCAGGTCTC
gBChE #3-F	CACCGTAGATCCATAGTGAAACGGT
gBChE #3-R	aaacACCGTTTCACTATGGATCTAC
gNt-F	CACCGTATTACTGATATTGGTGGG
gNt-R	AAACCCCACCAATATCAGTAATAC
**qRT-PCR**	
BChE-F	TCCATAGTGAAACGGTGGGC
BChE-R	AGGCCAGCTTGTGCTATTGT
β-Actin-F	TCACCATGGATGATGATATCGC
β-Actin-R	ATAGGAATCCTTCTGACCCATGC

**Table 2:** IC_50_ values (nM) of docetaxel (Dtx) and cabazitaxel (Cbz) in DU145 and 22Rv1 cells with shRNA or gRNA-mediated BChE knockdown compared to controls (shGFP and gNt). Data represent the effectiveness of taxane treatment across different depletion conditions (transcriptional suppression or genomic targeting).

Cell and # sh/gRNA	To Dtx (nM)	To Cbz (nM)
DU145 shGFP	2.25	2.05
DU145 shBChE #1	2.10	2.60
DU145 shBChE #2	8.53	11.02
DU145 shBChE #3	6.42	12.46
22Rv1 shGFP	7.50	7.66
22Rv1 shBChE #1	40.76	14.38
22Rv1 shBChE #2	19.28	16.10
DU145 gNt	2.74	2.03
DU145 gBChE #1	5.45	7.90
DU145 gBChE #2	4.73	4.63
DU145 gBChE #3	6.81	8.69
